# Structural Features of Highly Bioactive Citrus IntegroPectin Revealed by X‐Ray Diffraction, Fourier Transform Infrared, and Nanoparticle Tracking Analysis

**DOI:** 10.1002/open.70206

**Published:** 2026-04-20

**Authors:** Rosaria Ciriminna, Ana Rosa Garcia, Giuseppe Angellotti, Giovanna Li Petri, Chiara Valenza, Francesco Mauriello, Domenico Nuzzo, Pasquale Picone, Antonella Girgenti, Caterina Di Sano, Claudia D’Anna, Mario Pagliaro

**Affiliations:** ^1^ Istituto per lo Studio dei Materiali Nanostrutturati CNR Palermo Italy; ^2^ Centro de Química Estrutural Instituto Superior Técnico University of Lisbon Lisbon Portugal; ^3^ Department of Chemistry and Pharmacy Faculty of Science and Technology University of Algarve, Campus de Gambelas Faro Portugal; ^4^ Dipartimento di Ingegneria Civile, dell’Energia, dell’Ambiente e dei Materiali Università degli Studi Mediterranea di Reggio Calabria Reggio Calabria Italy; ^5^ Istituto per la Ricerca e l’Innovazione Biomedica CNR Palermo Italy; ^6^ Istituto per la Farmacologia Traslazionale CNR Palermo Italy

**Keywords:** citrus flavonoids, CytroCav, flavonoid‐pectin conjugate, IntegroPectin

## Abstract

Citrus IntegroPectin denotes a family of flavonoid‐pectin bioconjugates with large therapeutic potential sourced via the green extraction CytroCav process consisting of cavitation of industrial citrus processing waste conducted in water only, followed by dialysis and lyophilization or spray‐drying. We investigate the structure of lemon, orange, and red (blood) orange IntegroPectin using X‐ray diffraction, infrared spectroscopy, and nanoparticle tracking analysis. The analysis unveils several structural nuances unique to this new class of bioconjugates of relevance to forthcoming studies on the use of citrus IntegroPectin for therapy and prevention of numerous diseases.

## Graphical abstract

Structural investigation of lemon, orange, and red orange IntegroPectin using X‐ray diffraction, infrared spectroscopy, and nanoparticle tracking analysis unveils several structural nuances of relevance to forthcoming studies on the use of citrus IntegroPectin for therapy and prevention of numerous diseases.

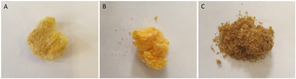

## 1 Introduction

Abundant in the cell wall of nongraminaceous plants and fruits (chiefly in the pericarp) where it acts as glue facilitating cell adhesion and separation but also modulates cell growth and shape [1], pectin is nature's structurally most complex polysaccharide [2]. Widely employed as the most valued food hydrocolloid by the food and beverage industry [3], its commercial uses are rapidly expanding also in several industrial sectors due to its unique physiological activity and versatility [4]. The heteropolysaccharide consists of a linear homogalacturonan (HG) polymer of *α*‐1,4‐D‐galacturonic acid (GalA) monomers, many of which are methyl‐esterified at O‐6 position (some also acetyl‐esterified at O‐2 or O‐3). The HG linear polymer is interrupted by branched rhamnogalacturonan‐I (RG‐I) regions composed of [→2)‐*α*‐L‐Rha‐(1→4)‐*α*‐D‐GalA‐(1→] repeats further binding neutral sugars including galactose, arabinose, xylose, and fructose, as well as by rhamnogalacturonan‐II (RG‐II) regions consisting of highly branched HG, with side chains at C‐2 and C‐3 including arabinose, apiose, fucose, galactose, rhamnose, aceric acid, glucuronic acid, galacturonic acid, xylose, and fucose [5].

Commercial production of pectin relies on hydrolysis of protopectin in dried citrus peel (a few plants start from fresh citrus peel) or apple pomace with hot dilute mineral acid at relatively high temperature [6]. Since then, this has become the industrial method to obtain a highly degraded form of the biopolymer which is extracted from dried citrus peels or apple pomace via prolonged hydrolysis promoted by dilute mineral acid (most often nitric acid) at relatively high temperature (70–80°C), followed by precipitation with isopropyl alcohol [7]. In general, pectin extracted via this process is high methoxyl (HM) pectin with the degree of esterification (DE) > 50%. Produced by controlled hydrolysis of HM pectin, low methoxyl (LM) pectin having DE < 50% is even more valued than HM pectin, because it gels without requiring sugar in a broad pH range in the presence of small amounts of Ca^2+^ ions.

Since the early 2000s, plentiful research efforts have been devoted to identify greener pectin extraction routes aimed at producing pectin with superior functional properties, including higher gel strength, better nutritional properties, and bioactivity [8]. Amid said new routes, the CytroCav process discovered in Italy [9] employs hydrodynamic (HC) or acoustic cavitation (AC) of industrial citrus processing waste (CPW) in water only, followed by freeze‐drying or spray‐drying. The method affords a new family of flavonoid‐pectin bioconjugates with large therapeutic potential named “IntegroPectin” [10]. Numerous in vitro and in vivo studies showed the multitarget biological activity of different citrus fruit (lemon, grapefruit, sweet orange, red orange, bitter orange, and mandarin) IntegroPectin, including immunomodulatory activity, antioxidant, anti‐inflammatory, cardioprotective, anti‐apoptotic, neuroprotective, mitoprotective, antimicrobial, and anticancer properties [10].

Said unique broad bioactivity has been ascribed to the unique molecular structure of these pectins having a LM HG backbone enriched in citrus flavonoids, terpenes, and RG‐I regions. The relative HG and RG‐I proportions control the polymer's flexibility and rheological behavior, with HG domains promoting intermolecular interactions, and branched RG‐I regions enhancing chain entanglement [11].

In brief, the complex structure of the IntegroPectin bioconjugates may generate a synergistic mechanism of action combining in a single treatment the bioactivity of citrus flavonoids with that of RG‐I enriched and LM pectin. The discovery of IntegroPectin, in other words, allows to merge the broad scope bioactivities of citrus flavonoids and RG‐I enriched pectin, and of highly bioactive LM pectin in particular [12], offering a synergistic solution to the poor bioavailability of flavonoids via their molecular binding to the heteropolysaccharide [13].

In this study, we combine different structural investigation techniques, including nanoparticle tracking analysis (NTA), to shed further light on the structure of IntegroPectin bioconjugates sourced from industrial CPW originating from different citrus fruits (lemon, sweet, and blood orange).

## 2 Results and Discussion

### 2.1 X‐Ray Diffraction Analysis

**FIGURE 1:**
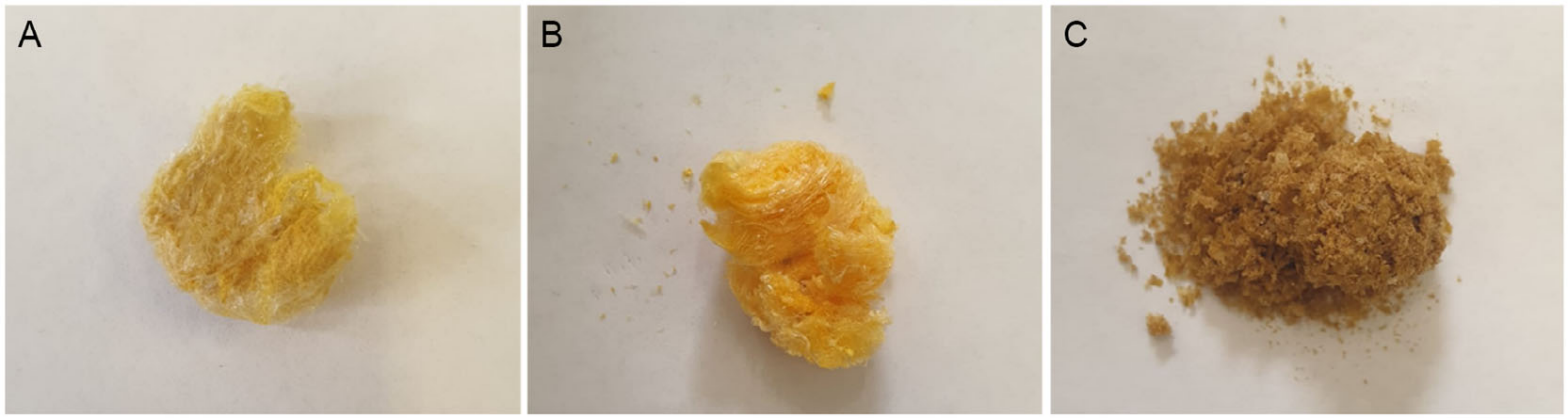
Citrus IntegroPectin samples sourced via AC from industrial CPW following obtained by freeze‐drying the precursor solution after 24 h dialysis: (A) lemon IntegroPectin, (B) sweet orange IntegroPectin, (C) red orange IntegroPectin. AC = Acoustic cavitation; CPW = citrus processing waste.

Samples of lemon, sweet orange, and red (blood) orange IntegroPectin sourced from industrial fresh CPW (derived from industrial squeezing fruits organically grown in Sicily) via AC were isolated in powder form (Figure 1) by freeze‐drying the corresponding aqueous extracts after 24 hr dialysis, as lately described [14].

**FIGURE 2:**
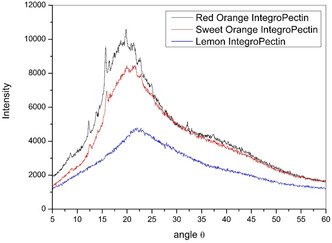
XRD patterns for the dialyzed IntegroPectin from red orange (black), sweet orange (red), and lemon (blue). XRD = X‐ray diffraction.

The X‐ray diffraction (XRD) patterns of the latter bioproducts are shown in Figure 2. It should be noted that in the case of pectin, only the diffraction from the HG regions contributes to the XRD pattern [15]. In contrast to commercial citrus pectin showing diffraction peaks centered at 13.65° and 21.26° related to second‐order reflections of helical structure of the HG chain [16] along with other small but sharp diffraction peaks characteristic of commercial citrus pectin at 12.4°, 14.3°, 21.0°, 28.9°, 31.5°, 32.2°, and 40.2° [17], the former two peaks in Figure 1 are shifted to higher 2*θ* values, and several peaks disappear.

This behavior is similar to that observed in the case of lemon and grapefruit IntegroPectin bioconjugates sourced via HC in water only, with flavonoids reducing the degree of crystallinity, and shifting both first and second diffraction peaks [18]. Said change indicates that AC of citrus biowaste, likewise to HC, induces partial reduction in crystallinity of the homogalacturonan chains destructuring the “fringed‐micellar” regions of the semicrystalline pectin biopolymer [19]. In other words, cavitation, and not the presence of acid or base, is responsible for the reduction in crystallinity of pectin observed in IntegroPectin. Remarkably, a similar finding has been reported also in the case of pectin extracted via AC of dried grapefruit peels at pH 1.5 and 67°C [20].

This outcome explains the significantly larger solubility of IntegroPectin sourced via the CytroCav process in water at room temperature when compared to the poorly soluble commercial citrus pectin. Amorphous polysaccharide with chains and their segments in amorphous regions disordered or disorganized leave unsatisfied hydrogen‐bonding positions to hydrate when dissolved in water, leading to rapid dissolution rate and solubility [21]. Crystallinity, on the other hand, prevents water penetration and consequent polymer dissolution, as free hydrogen bonds are not available for hydration [22].

### 2.2 Zeta‐Potential and Nanoparticle Tracking Analyses

The particle HC diameter, concentration, and ζ‐potential of the three IntegroPectin bioconjugates dissolved in water were measured by dynamic light scattering with a 488 nm laser in scatter mode and a high‐sensitivity camera.

Results in Table 1 indicate that particle sizes were comparable, ranging from 339 nm for sweet orange IntegroPectin to 399 nm for lemon IntegroPectin. For comparison, the hydrodynamic diameter (HC) of commercial citrus pectin amounts to 323 nm. The polydispersity index (PDI) of the IntegroPectin bioconjugates investigated was low, ranging from 0.210 for sweet orange IntegroPectin to 0.344 for the lemon‐derived bioconjugate.

**TABLE 1:** HC diameter, PDI, and ζ‐potential were measured by dynamic light scattering analysis.

Pectin	***D***_**H**_ **, nm**	PDI	ζ‐potential, mV
Lemon IntegroPectin	399 ± 151	0.344	−37 ± 6.03
Commercial citrus pectin	323 ± 144	0.324	−24.1 ± 3.92
Sweet orange IntegroPectin	339 ± 147	0.210	−26.1 ± 3.91

**FIGURE 3:**
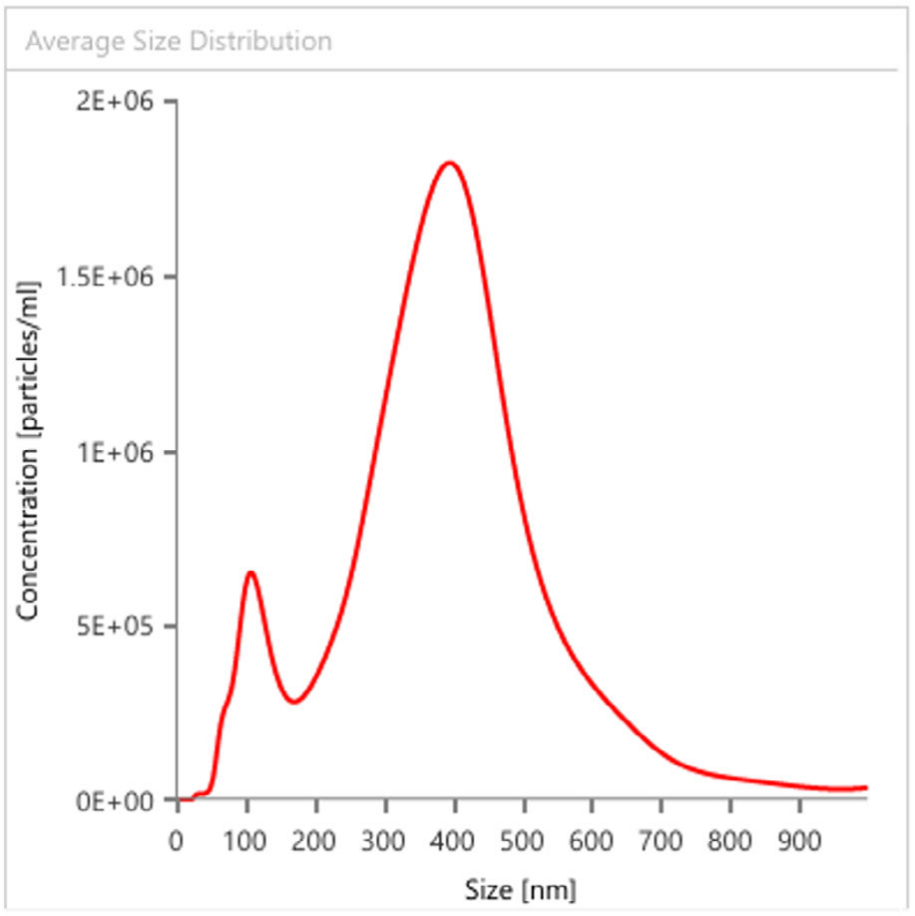
Average particle size distribution for commercial citrus pectin.

Representing the particle distribution width, the 1.39 Span value (*d*_90_ *–d*_10_)/*d*_50_, where *d*_90_, *d*_50_, and *d*_10_ are the particle diameters at 90%, 50%, and 10% of cumulative volume for commercial citrus pectin, indicates low polydispersity, namely a polysaccharide with a relatively narrow distribution (Figure 3) having two peaks: one at ca. 400 nm and another, one order of magnitude less abundant, at 110 nm. This is consistent with the presence of relatively large polymeric pectin molecules originating from the polysaccharide extracted with dilute mineral acid after dissolved in water.

**FIGURE 4:**
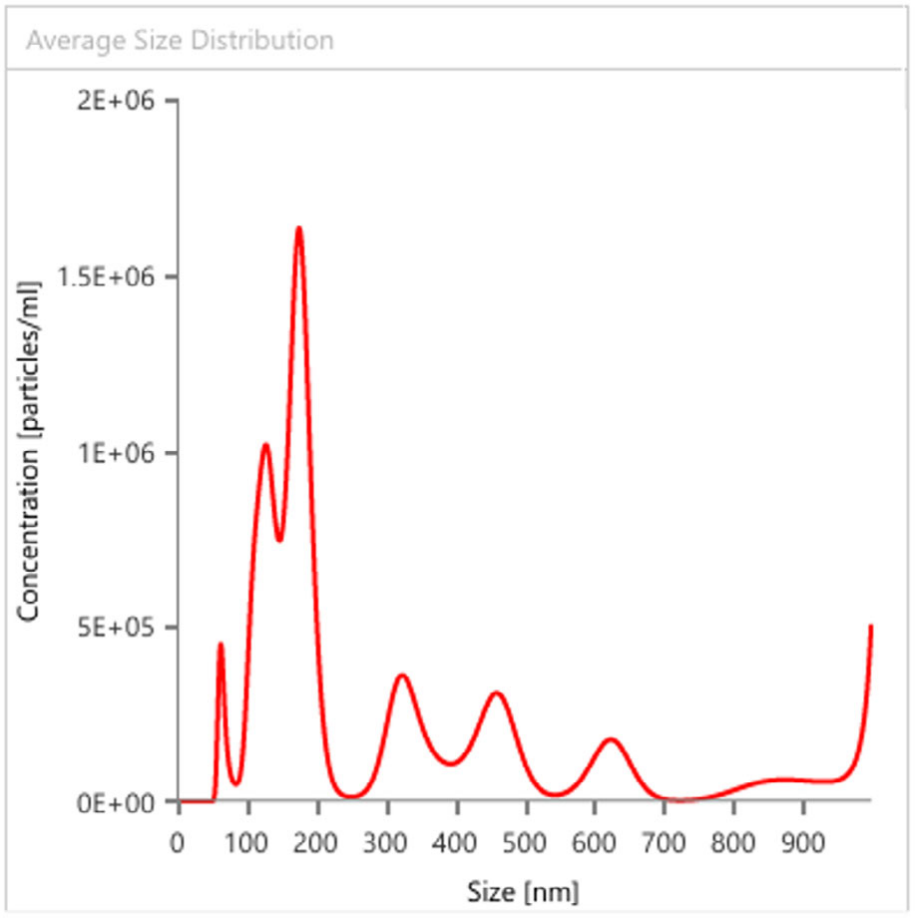
Average particle size distribution for lemon IntegroPectin.

In the case of lemon IntegroPectin (total concentration of particles, 1.93x10^11^ per mL of sample analyzed), the 2.65 Span value indicates substantially higher polydispersity with the most frequent particle size being ≈176.5 nm, followed by a second most abundant population at 150 nm (Figure 4).

**FIGURE 5:**
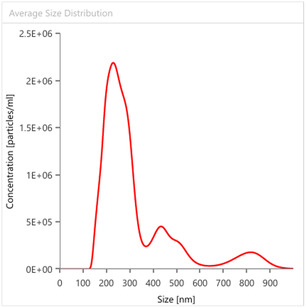
Average particle size distribution for sweet orange IntegroPectin.

Sweet orange IntegroPectin (total concentration of particles, 3.93x10^11^ per mL of sample analyzed), too, has low Span value (1.57) indicating low polydispersity with the most frequent particle size being ≈229.5 nm (Figure 5).

Finally, the IntegroPectin phytocomplexes derived from lemon and sweet orange had high negative values of ζ‐potential (−37 and −26.1 mV, respectively), substantially higher than that of the of commercial citrus pectin (−24.1 mV). This indicates the higher esterification degree imparted by the concomitant presence of citric acid and pectin during the cavitation‐based extraction process from fresh CPW in comparison to commercial citrus pectin typically sourced from dried citrus peel via the conventional acid‐assisted extraction [6]. The higher amount of citric acid present in lemon processing waste affords the highest ζ‐potential observed in the case of lemon IntegroPectin due to higher extent of pectin esterification with citrate groups.

These findings indicate that cavitation of CPW in water only at room temperature (the CytroCav process) affords pectin particles of substantial lower size when compared to commercial citrus pectin sourced with mineral acid in hot water via the conventional industrial process. Citric acid seems to impact the particle size distribution affording smaller particles but with a more polydisperse distribution. This is in agreement with recent findings for which the extraction of pectin from dried peels of *Citrus reticulata* with citric acid results in RG‐I enriched pectin having a highly branched structure and shorter side chains facilitating intramolecular hydrophobic interactions [23]. For comparison, whereas the CytroCav process directly affords pectin particles in solution having HC diameter between 323 and 399 nm, similar reduction in size of commercial citrus pectin extracted with the conventional extraction process required five steps [24]. Said five steps (extraction with mineral acid followed by microwave treatment, high‐speed homogenization, ultrasonic treatment and spray‐drying) eventually reduced the size of untreated pectin particles from 1402 nm to less than 300 nm.

**FIGURE 6:**
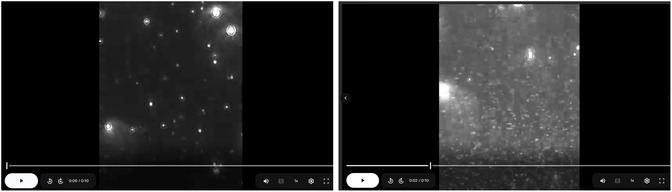
Frames of nanoparticle tracking videos for sweet orange IntegroPectin (left) and lemon IntegroPectin (right) in aqueous solution. Clicking on the image allows online access to linked videos at, respectively, https://t.ly/H2dPL for sweet orange and https://t.ly/Uu9Jo for lemon IntegroPectin.

Videos of the NTA experiment clearly show the submicron IntegroPectin particles. Recorded videos too show evidence (thumbnails in Figure 6) that the particles of sweet orange and lemon IntegroPectin are similar, with lemon bioconjugate particles being somewhat smaller, and with broader particle size distribution evidenced by the concomitant appearance of smaller and larger particles.

Further evidence of the relevance of nanoscale morphology for pectin's bioactivity was lately reported by comparing the activity of submicron (278 nm) pectin particles of spherical shape (obtained after conventional acid hydrolysis followed by microwave, homogenizer, ultrasound treatment, and spray‐drying) with that of conventional pectin in reducing all consequences of increased oxidative stress in rats [24]. The new “nano‐pectin” turned out to be significantly more active than that of citrus pectin sourced via conventional acid hydrolysis.

### 2.3 Infrared Spectroscopy Analysis

**FIGURE 7:**
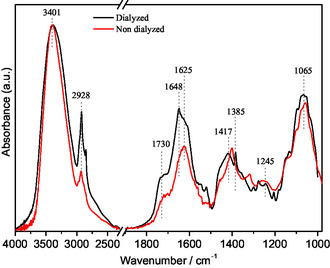
Infrared spectra of IntegroPectin samples from red orange, dialyzed and nondialyzed as indicated, normalized to the maxima absorption.

Figure 7 compares the spectra of two IntegroPectin bioconjugates obtained from red orange samples, one dialyzed and the other nondialyzed, highlighting some differences in their overall band structures. The infrared spectrum of the nondialyzed citrus pectin sourced via microwave‐assisted extraction in water only has been thoroughly analyzed in previous studies [25].

The νO–H band, with maxima at ≈3401 cm^−1^ for both dialyzed and nondialyzed samples, is associated with hydroxyl groups of the pyranose rings and with adsorbed water involved in different intra‐ and intermolecular hydrogen‐bonding interactions, apparently very similar in the two samples. The band broadening to lower wavenumbers appears to indicate a higher amount of stronger hydrogen bonding in the dialyzed IntegroPectin sample. The band with a maximum at 2928 cm^−1^ consists of a set of overlapping modes assigned to νCH and ν_as_CH_3_ vibrations of the pectin backbone, as well as to ν_as_CH_2_ modes of galactose and arabinose rings.

Related with the carbonyl stretching mode, in the 1800–1500 cm^−1^ region, the spectra of both IntegroPectin samples present two bands with maxima near 1730 and 1625 cm^−1^, which can be assigned to the stretching vibrations of carbonyl groups, mainly from esterified galacturonic acid: ν(C=O) of the ester group, and ν_as_(COO^–^) of carboxylate groups, respectively. The main CH_*x*_ and C–O–H deformation modes appear partially overlapped in the 1500–1200 cm^−1^ region. As typically observed for pectin, several intense and partially overlapped bands are present in the 1200–950 cm^−1^ region. These are attributed to skeletal and C–O–C stretching modes of the pyranose ring (ν(C–C) and ν(C–O–C)), to C–O–C stretching vibrations of the glycosidic bond, and to combinations of ν(C–OH) and ν(C–C) modes from the pyranose rings. A more complete band assignment for the dialyzed IntegroPectin sample is shown in Table 1.

It is possible that the IntegroPectin samples also contain flavonoids, as well as sugars such as glucose, fructose, and sucrose, and citric acid. The most informative bands of these compounds are as follows:

‐ For flavonoids, strong infrared bands associated with ν(OH), ν(C=O), and ν(C=C) vibrations of the aromatic ring commonly appear in the 3400–3200 cm^−1^, 1600 ± 100 cm^−1^, and 1600–1300 cm^−1^ ranges, respectively, depending on the flavonoid structure [26, 27].
‐ The infrared spectra of sugars present two characteristic regions: 3500–2700 cm^−1^, showing intense bands related to ν(OH) and ν(CH_*x*_) modes, and the more intense 1200–900 cm^−1^ region, containing a complex sequence of strong bands arising from highly coupled C–C, C–O stretching and C–O–H, C–O–C deformation modes of various oligo‐ and polysaccharides. If the three sugars are present in the IntegroPectin samples, the intense and broad band in the low‐frequency region with a maximum between 1060 and 1020 cm^−1^ is the one that can be influenced by bands at 1053 cm^−1^ for fructose, 1049 cm^−1^ for sucrose, and 1032 cm^−1^ for glucose [28, 29].
‐ For citric acid, in the infrared spectrum may be observed the vibrational modes at ≈3440 cm^−1^ (ν(OH) of alcohol groups), 3340–3000 cm^−1^ (ν(OH) of acid groups), 1800–1730 cm^−1^ (ν(C=O) of ester carbonyls), and 1750–1650 cm^−1^ (ν(C=O) of acid carbonyls) [30].

Based on above considerations, the main bands of the compounds that may be present alongside pectin are, in most cases, superimposed on or strongly overlapped with those of pectin, making it difficult to attribute all spectral changes observed between the nondialyzed and dialyzed IntegroPectin samples to the presence or absence of any individual species. Nevertheless, the bands assigned to ν(CH_*x*_) modes of pectin appear better defined and relatively stronger in the dialyzed sample, accompanied by a similar behavior of the δ_as_(CH_3_) mode at 1648 cm^−1^. In the low wavenumber region, it is very clear a better band definition in the dialyzed sample, particularly in the 1500–1200 cm^−1^ region (mainly CH_*x*_ and C–O–H deformation modes); nevertheless due to complexity and extensive band overlap, drawing definitive conclusions is more challenging.

**FIGURE 8:**
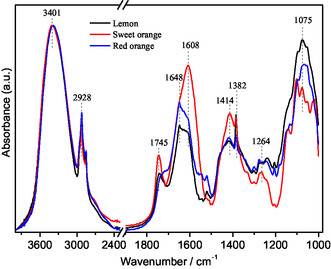
Infrared spectra of dialyzed IntegroPectin samples, normalized to the maxima absorption, obtained from lemon, sweet orange, and red orange, as indicated.

The infrared spectra of the dialyzed IntegroPectin samples obtained from lemon and from two orange varieties (sweet and red) are shown in Figure 8. The immediate conclusion is that the overall band structure is similar across the three samples, although clear differences in their relative intensities are observed.

The broad ν(OH) region for all IntegroPectin samples exhibits a strong and broad absorption band near 3401 cm^−1^, associated with hydroxyl groups of the pyranose rings and with adsorbed water involved in different intra‐ and intermolecular hydrogen‐bonding interactions [25]. The profiles closely overlap, suggesting similar water content and comparable amounts of hydroxyl‐containing compounds. The band centered at 2928 cm^−1^, associated with C–H stretching modes, appears slightly sharper in the sample from lemon, whereas the two samples from orange show similar profiles.

Complete band assignment based on infrared analysis of pectins sourced from the peels of different citrus fruits suspended in water only (no added acid) using microwaves [25] is shown in Table 2.

**TABLE 2:** Assignment of the Infrared spectra bands of dialyzed IntegroPectin samples, obtained from lemon, sweet orange, and red orange.

Band position of the IntegroPectin sample, $\tilde{\nu }$ **, cm** ^**−1**^			
Lemon	Sweet orange	Red orange	Band assignment
3401	3401	3387	νOH
2961_(sh)_	2955_(sh)_	2955_(sh)_	νCH
2928	2928	2928	
2854	2854	2853	ν_s_CH_3_; pyranose rings
1741	1745	1735_(sh)_	νC=O; overlapped of carboxylic acid and ester modes
1720_(sh)_	1720_(sh)_	1720_(sh)_	
1648_(br)_		1648_(br)_	δ_as_CH_3_; ester
	1608_(br)_		ν_as_COO−; overlapped with νC=C of flavonoids
	1520	1524_(sh)_	
1420	1414	1419	δ_as_CH_3_; ester
1384	1385	1385	δ_s_CH_3_;
	1325_(sh)_		δ_s_CH_3_; ester
1301	1301	1301	δ_s_CH_3_;
1275	1275_(sh)_	1277	νC–O–C; ester
1261	1265		
1240	1242_(sh)_	1244	νC–O
1206	1206	1206	
		1181	
	1142	1147_(sh)_	νC–O–C; pyranose mode
1133_(sh)_		1132	
1095	1102	1095	Combination bands νC–OH + νC–C; pyranose modes
1075	1077	1077_(sh)_	
		1067	
1054	1053	1054_(sh)_	νC–O–C; glycoside mode
1030_(sh)_	1026_(sh)_	1035_(sh)_	νC–O–C + νC–C; pyranose modes
1015_(sh)_	1017		

*Note:* sh—shoulder.

In the 1800–1500 cm^−1^ region, the band at ≈1745 cm^−1^, with a shoulder at ≈1720 cm^−1^, associated with C=O stretching in esters or organic acids, is stronger in the sweet orange IntegroPectin, suggesting a higher content of ester and/or acid groups. The bands with relative maxima at 1648 and 1608 cm^−1^, assigned to ν_as_(COO^–^) and overlapping with ν(C=C) of flavonoids, are strongly sample dependent. In the sample from sweet orange, the maximum appears at 1608 cm^−1^, whereas in those from lemon and red orange, the maxima are at 1648 cm^−1^ with a shoulder at 1608 cm^−1^, and the relative intensity is clearly lower.

The C–H bending modes with maxima at 1414 and 1382 cm^−1^, assigned to δ_as_(CH_3_) of esters and δ_as_(CH_3_) of other molecules, respectively, exhibit similar profiles, with the band at 1382 cm^−1^ showing slightly higher intensity. A more pronounced difference is observed in the ν(C–O–C) mode of the ester region at 1264 cm^−1^, indicating possible variations in carbohydrate or pectin structures. A similar conclusion can be drawn from the lowest wavenumber region, where the strong band centered at ≈1075 cm^−1^ results from a set of overlapping bands, including ν(C–OH) + ν(C–C) combination bands, ν(C–O–C) of glycosides, and ν(C–O–C)+ν(C–C) pyranose combination modes.

The C–H bending modes with maxima at 1414 and 1382 cm^−1^, assigned to δ_as_(CH_3_) of esters and δ_as_(CH_3_) of other molecules, respectively, have a similar profile with a slightly higher intensity of the band at 1382 cm^−1^. A clearer difference is observed in the ν(C–O–C) mode of ester group region, 1264 cm^−1^, indicating a possible change in the carbohydrate or pectin structures. The same conclusion can be drawn from the lowest wavenumber region, from the strong band centered ≈1075 cm^−1^, that results from a set of overlapped bands, ν(C–OH) + ν(C–C) combination bands, ν(C–O–C) of glycosides and ν(C–O–C) + ν(C–C) pyranose combination modes.

To refine the interpretation of the infrared spectra, spectral deconvolution was performed in the 1800–1530 cm^−1^ and 1195–1000 cm^−1^ regions. This approach allowed us to resolve overlapping bands and obtain a more accurate assignment of the vibrational modes. Band positions and areas were estimated by decomposing each region into a sum of Gaussian components using a nonlinear least‐squares fitting method.

Table 3 compares the dialyzed IntegroPectin samples (from lemon, red orange, and sweet orange) with the nondialyzed IntegroPectin (from red orange) focusing on characteristic vibrational bands and semiquantitative compositional indicators derived from band deconvolution.

**TABLE 3:** Summary of the results obtained by deconvolution of the infrared spectra in the 1800–1530 and 1195–1000 cm^−1^ regions: wavenumber ($\tilde{\nu }$, cm^−1^); A—integrated area.

	Lemon (dialyzed)		Red orange (dialyzed)		Sweet orange (dialyzed)		Red orange (nondialyzed)	
Assignment	$\tilde{\nu }$, cm^−1^	A	$\tilde{\nu }$, cm^−1^	A	$\tilde{\nu },$cm^−1^	A	$\tilde{\nu }$, cm^−1^	A
ν(C=O)_methyl‐ester_	1746	2.17	1738	4.19	1745	4.24	1740	0.53
νC=O)_ester_	1722	7.13	1712	2.05	1713	3.84	1711	4.48
ν(C=O)_citric acid_	1678	2.51	1676	7.21	1679	6.38	1652	7.14
ν(C=O)_carboxylic acid_	1652	5.90	1651	3.42	1653	19.05	1617	8.49
ν_as_(COO^−^) + ν(C=C)_flavenoids_	1614	15.43	1620	15.98	1610	9.52	1572	3.14
ν(C–O–C)_pyranose_	1143	7.58	1140	7.17	1145	8.81	1140	6.59
ν(C–O–C)_pyranose_	1097	11.72	1098	7.94	1103	11.81	1100	6.74
ν(C–O–C)_pyranose_	1075	2.54	1076	3.92	1075	5.66	1080	2,18
ν(C–O–C)_glycoside_	1053	9.29	1054	10.37	1051	7.00	1062	5.80
ν(C–O–C)_glucose_	1026	2.44	1026	3.94	1026	4.10	1036	13.78
ν(C–OH)_pyranose_ +ν(C–C)_pyranose_	1012	0.40	1009	0.95	1012	2.52	986	2.99
Based on components retrieved by band deconvolution, estimative of:								
DE (%)	‐‐‐‐‐	28	‐‐‐‐‐	19	‐‐‐‐‐	19	‐‐‐‐‐	21
HG (∝ to)	‐‐‐‐‐	0.39	‐‐‐‐‐	0.39	‐‐‐‐‐	0.37	‐‐‐‐‐	0.38
Flavonoids (∝ to)	‐‐‐‐‐	0.47	‐‐‐‐‐	0.49	‐‐‐‐‐	0.22	‐‐‐‐‐	0.13
Citric acid (∝ to)	‐‐‐‐‐	0.08	‐‐‐‐‐	0.22	‐‐‐‐‐	0.15	‐‐‐‐‐	0.30
Glucose (∝ to)	‐‐‐‐‐	0.07	‐‐‐‐‐	0.11	‐‐‐‐‐	0.10	‐‐‐‐‐	0.36
Sugars + Glycosides (∝ to)	‐‐‐‐‐	0.35	‐‐‐‐‐	0.42	‐‐‐‐‐	0.28	‐‐‐‐‐	0.51

*Note:* DE, percentage of the DE; HG, proportional amount of galacturonic acid‐rich; Citric acid—proportional amount of citric acid; Glucose—proportional amount of glucose; Sugars + Glycosides—proportional amount of other sugars and glycosides.

Based on the results of the band deconvolution, it is possible to examine in more detail the structure of the pectins, their DE, and the presence of flavonoids, citric acid, sugars, and glycosides in the IntegroPectin samples.

The percentage of esterified carboxyl groups, the DE, can be estimated from the ratio of the ester carboxyl to the total carboxyl band areas, using Equation (1) [31, 32]. This calculation relies on the component bands obtained from the spectral analysis of the 1500–1800 cm^−1^ region.

(1) $$\mathrm{DE}=\frac{\sum A_{\nu C=O(\text{ester})}}{\sum A_{\nu C=O(\text{ester})}+\sum A_{\nu C=O\left(\text{acid}\right)}+\sum A_{\nu \mathrm{asC}{\mathrm{OO}}^{-}}}\times 100$$

As noted in the introduction, pectin is composed primarily of homogalacturonan (HG) and rhamnogalacturonan I (RG‐I) segments, whose relative proportions determine the polymer's flexibility and rheological properties. Although the absolute amounts of HG regions cannot be calculated, they are proportional to the ratio defined in Equation (2), obtained from the components retrieved from both spectral regions [33].

(2) $$\mathrm{HG}\propto \frac{\sum A_{\nu C=O}+\sum A_{\nu \text{asCO}O^{-}}}{\sum A_{\nu C=O}+\sum A_{\nu \text{asCO}O^{-}}+\sum A_{\nu \mathrm{COC}}+\sum A_{\nu \mathrm{COH}/\nu \mathrm{CC}}}$$

Similarly, the amounts of flavonoids, citric acid, and sugars cannot be calculated directly, as for HG. However, their relative contributions can be inferred by considering that certain infrared bands are particularly sensitive to, or characteristic of, these components within the samples. Using the established characteristic band positions of flavonoids [26, 27], citric acid [30], glucose [28], and sugars + glycosides [29], their relative contributions can be expressed through the ratios defined in Equations (3), (4), (5), and (6), respectively.

(3) $$\text{Flavonoids}\propto \frac{A_{\nu \text{asCO}O^{-}/\nu C=C}}{\sum A_{\nu C=O}+A_{\nu \text{asCO}O^{-}/\nu C=C}}$$

(4) $$\text{Citric acid}\propto \frac{A_{\nu C=O_{\left(\text{citric acid}\right)}}}{\sum A_{\nu C=O}+A_{\nu \text{asCO}O^{-}/\nu C=C}}$$

(5) $$\text{Glucose}\propto \frac{A_{\nu {\left(\mathrm{COC}\right)}_{\text{glucose}}}}{\sum A_{\nu \mathrm{COC}}+\sum A_{\nu \mathrm{COH}/\nu \mathrm{CC}}}$$

(6) $$\text{Sugars}+\text{Glycosides}\propto \frac{A_{\nu {\left(\mathrm{COC}\right)}_{\text{glucose}}}+A_{\nu {\left(\mathrm{COC}\right)}_{\text{glycoside}}}}{\sum A_{\nu \mathrm{COC}}+\sum A_{\nu \mathrm{COH}/\nu \mathrm{CC}}}$$

The infrared spectra deconvolution suggests that the dialyzed IntegroPectin samples from lemon and red orange exhibit similar profiles, characterized by elevated flavonoid and sugar/glycoside contributions, reflecting a more heterogeneous matrix.

Considering the estimated values for the DE, it is possible to infer structural differences among the IntegroPectin samples. IntegroPectin samples from red orange and sweet orange show similar DE values (19%), whereas lemon IntegroPectin exhibits a higher DE (28%), indicating a more esterified structure that can be justified by a higher amount of citric acid present in lemon processing waste. It should be noted that esterification of pectin with flavonoids in the case of IntegroPectin has been lately forecasted based on density functional theory calculations [34] and subsequently confirmed experimentally in China in the case of pectin in orange peel found partly conjugated with hesperitin [35].

The proportion of homogalacturonan, the primary smooth‐region domain of pectin, is very similar amid all samples. IntegroPectin samples from lemon and red orange show similar HG proportions (0.39), while the sweet orange sample exhibits a slightly lower but comparable value (0.37).

With respect to the relative amounts of flavonoids and sugars, the IntegroPectin samples display opposite trends. Flavonoid‐related contributions are highest in the IntegroPectin samples obtained from lemon and red orange (0.47 and 0.49). The sweet orange sample shows the lowest flavonoid contribution (0.22). This is in agreement with the substantially higher amount of citric acid present in lemon and red orange biowaste [36], with acid needed to promote the esterification reaction between carboxylate groups in pectin and flavonoids.

In contrast, sugars and glycosides follow an inverse pattern: lemon and red orange exhibit higher contributions compared with sweet orange. All samples show higher sugar + glycoside proportions (lemon: 0.35; red orange: 0.42), while the sweet orange sample has a lower value (0.28).

Comparison of the deconvoluted spectra from dialyzed and nondialyzed IntegroPectin samples indicates that the DE and the proportion of homogalacturonan regions remain unaffected by the dialysis process. However, the data also reveal an increased relative abundance of flavonoids, accompanied by a reduction in the proportions of citric acid, sugars, and glycosides.

These observations are consistent with the selective removal of low‐molecular‐weight and highly water‐soluble compounds during dialysis. The DE and the relative proportion of homogalacturonan domains are primarily determined by the polysaccharide backbone, which is not expected to diffuse through the dialysis membrane and therefore remains unchanged. In contrast, citric acid, simple sugars, and small glycosylated molecules possess molecular sizes and solubility characteristics that favor their removal during dialysis, explaining their reduced spectral contributions in the dialyzed samples. Conversely, flavonoids, often present as larger, more hydrophobic, or polymer‐associated species, tend to be retained, resulting in an apparent increase in their relative abundance in the deconvoluted spectra.

## 3 Conclusions

In summary, structural investigation via XRD, infrared spectroscopy, and NTA of lemon, sweet, and red orange IntegroPectin sourced via AC of fresh industrial CPW conducted in water only, and isolated via freeze‐drying following dialysis against water, reveals several structural aspects of relevance to future research on IntegroPectin for therapy and prevention of disease.

First, cavitation of CPW in water only at room temperature (the CytroCav process) affords more polydisperse pectin particles of lower size when compared to commercial citrus pectin, with residual citric acid in CPW promoting the formation of more esterified pectic polymer having higher (more negative) ζ‐potential. Nanoscale morphology of pectin hydrocolloid submicron particles is of direct relevance to pectin's bioactivity, with the activity of spherical submicron particles being substantially higher than that of conventional citrus pectin.

Second, likewise to what happens with IntegroPectin bioconjugates sourced via HC cavitation, also the IntegroPectin bioconjugates sourced via AC are LM pectins, with red orange and sweet orange conjugates showing similar DE values (19%), and lemon IntegroPectin exhibiting a higher DE (28%).

Third, the proportion of homogalacturonan, the primary smooth‐region domain of pectin, is very similar amid all samples. IntegroPectin samples from lemon and red (blood) orange show similar HG proportions (0.39), while the sweet orange sample exhibits a slightly lower but comparable value (0.37).

Fourth, citric acid abundant in lemon and red orange biowaste plays an important role in enhancing conjugation of pectin and citrus flavonoids.

Of direct relevance to forthcoming pharmaceutical and nutraceutical applications of citrus IntegroPectin bioconjugates, these findings are important also for forthcoming applications of IntegroPectin in producing pectin‐based gels and films in regenerative medicine.

## 4 Experimental Section

### 4.1 Materials

Each IntegroPectin sample was obtained as lately described via AC, followed by dialysis and freeze‐drying [14]. In brief, an aliquot (300 g) of CPW originating from different citrus fruits (lemon, sweet orange, and red (blood) orange) was added with 3 L of ultrapure water obtained using a Barnstead Smart2Pure Water Purification System (Thermo Fisher Scientific, Waltham, MA, USA). The mixture was homogenized with a domestic electric blender by grinding twice for 30 s at high speed each time. Extraction of IntegroPectin was conducted using the UIP2000hdT (20 kHz, 2000 W) industrial sonicator (Hielscher Ultrasonics, Teltow, Germany) equipped with a hydraulic pump setting the flow at 1.43 L/min to carry out the process in continuous flow mode for 30 min at 50% of amplitude in pulse condition (50 s on–50 s off). Power supplied to the digital probe‐type sonicator was set at 800 W. Maximum work temperature for the mixture is at 50°C. After extraction was complete, the mixture was filtered through a cotton cloth to separate the insoluble fraction from the aqueous phase. The aqueous phase containing the IntegroPectin in solution was further filtered through a Büchner funnel using filter paper (Whatman, grade 589/3, retention <2 μm). Each IntegroPectin sample was dissolved in 5 mL ultrapure water followed by dialysis through a commercial dialysis bag (12 kDa cut‐off, Millipore, USA) immersed in 50 mL ultrapure water. After the first hour, the whole acceptor fluid was replaced with fresh ultrapure water. Eventually, IntegroPectin was isolated by freeze‐drying using a FreeZone 4.5 Liter Benchtop Freeze Dry System (Labconco, Kansas City, MO, USA). Fourier transform infrared (FTIR) grade KBr (≥99% purity) for the infrared experiments was purchased from Sigma‐Aldrich (St. Louis, MO, USA).

### 4.2 X‐Ray Diffraction Analysis

XRD patterns were collected using a Bruker AXS D5005 diffractometer (Karlsruhe, Germany) operated at 40 kV and 30 mA. Data were recorded over the 2*θ* range from 5° to 60° at a scan speed of 0.15°·min^−1^. The instrument employed Cu K*α* radiation, with monochromation provided by the secondary monochromator.

### 4.3 Dynamic Light Scattering Analysis

Each IntegroPectin sample was dispersed in ultrapure water at 1 mg·mL^−1^. HC size distributions and ζ‐potential were measured on a Zetasizer Nano ZS (Malvern Panalytical, Malvern, Great Britain). Reported values correspond to the mean of three measurements.

### 4.4 Nanoparticle Tracking Analysis

Particle size and concentration were analyzed using a NanoSight Pro instrument (Malvern Panalytical) with a 488 nm laser in scatter mode and a high‐sensitivity sCMOS camera. The data were processed using NS Explorer Build 1.1.0.6 software. For analysis, the samples were diluted (dilution factor 1:1000) in particle‐free water to obtain an optimal concentration within the recommended measurement range (1–10 × 10^8^ particles/mL). The instrument was configured according to the manufacturer's guidelines. High video quality was set for the analysis with a frame rate of 30 frames/s. The acquisition duration was set to 750 frames for five analyses for each sample. The results are expressed using the FTLA model, which groups particles of similar sizes into different distribution ranges.

### 4.5 Fourier Transform Infrared Analysis

The FTIR spectra were recorded using a FTIR spectrometer (Bruker, Billerica, MA, USA) equipped with OPUS 7.0 software, spanning the 4000–400 cm^−1^ range, with a lateral resolution of 2 cm^−1^ and 128 scans. For sample preparation, ≈1 mg of lyophilized IntegroPectin was finely mixed with 100 mg of KBr in a mortar until a homogeneous powder was obtained. The mixture was then compacted using a Specac Mini‐Pellet laboratory hydraulic press operated at 12 t for 5 min to obtain transparent KBr pellets suitable for spectroscopic analysis. The infrared data were processed using the Origin software (OriginLab Corporation, Northampton, MA, USA).

## Funding

R.C. and M.P. thank the Ministero dell’Università e della Ricerca for funding, progetto “FutuRaw. Le materie prime del futuro da fonti non‐critiche, residuali e rinnovabili,” Fondo Ordinario Enti di Ricerca, 2022, CNR (CUP B53C23008390005). D.N. and P.P thank Ministero dell’Università e della Ricerca for funding, project “Emerging Infectious Diseases One Health Basic and Translational Research Actions addressing Unmet Needs on Emerging Infectious Diseases,” INF‐ACT, Spoke 1 and Spoke 5, funded by the European Union NextGenerationEU (PNRR – project number PE00000007, CUP B53C20040570005). Work of G.L.P. was financially supported by the Made in Italy ‐ Circular and Sustainable (MICS) Extended Partnership funded by the European Union NextGenerationEU (PNRR – Missione 4, Componente 2, Investimento 1.3 – D.D. 1551.11–10–2022, PE00000004). Work of G.A. was financially supported by the SAMOTHRACE (Sicilian Micro and Nano Technology Research and Innovation Center) Innovation Ecosystem funded by the European Union NextGenerationEU (PNRR – Mission 4 Component 2 – Investment 1.5 (ECS00000022) – CUPB63C22000620005).

## Conflicts of Interest

The authors declare no conflicts of interest.

## Data Availability

All data are available upon reasonable request by contacting the corresponding authors.
